# Toward the Manufacturing of a Non-Toxic High-Performance Biobased Epoxy–Hemp Fibre Composite

**DOI:** 10.3390/polym16142010

**Published:** 2024-07-13

**Authors:** Gilles Boni, Vincent Placet, Marina Grimaldi, Patrick Balaguer, Sylvie Pourchet

**Affiliations:** 1 Institut de Chimie Moléculaire Université de Bourgogne (ICMUB), UMR 6302, 21000 Dijon, France; gilles.boni@u-bourgogne.fr; 2 Institut FEMTO-ST, CNRS, Université de Franche-Comté, 25000 Besançon, France; vincent.placet@univ-fcomte.fr; 3 Institut de Recherche en Cancérologie de Montpellier (IRCM), INSERM U1194, Université de Montpellier, Institut Régional du Cancer de Montpellier (ICM), 34090 Montpellier, France; marina.grimaldi@icm.unicancer.fr (M.G.); patrick.balaguer@inserm.fr (P.B.)

**Keywords:** biobased epoxy, plant fibre composites, mechanical properties, DMA, viscosity

## Abstract

This study describes the production of a new biobased epoxy thermoset and its use with long hemp fibres to produce high-performance composites that are totally biobased. The synthesis of BioIgenox, an epoxy resin derived from a lignin biorefinery, and its curing process have been optimised to decrease their environmental impact. The main objective of this study is to characterise the rheology and kinetics of the epoxy system with a view to optimising the composite manufacturing process. Thus, the epoxy resin/hardener system was chosen considering the constraints imposed by the implementation of composites reinforced with plant fibres. The viscosity of the chosen mixture shows the compatibility of the formulation with the traditional implementation processes of the composites. In addition, unlike BPA—a precursor of diglycidyl ether of bisphenol A (DGEBA) epoxy resin—BioIgenox and its precursor do not have endocrine disrupting activities. The neat polymer and its unidirectional hemp fibre composite are characterised using three-point bending tests. Results measured for the fully biobased epoxy polymer show a bending modulus, a bending strength, a maximum strain at failure and a T_g_ of, respectively, 3.1 GPa, 55 MPa, 1.82% and 120 °C. These values are slightly weaker than those of the DGEBA-based epoxy material. It was also observed that the incorporation of fibres into the fully biobased epoxy system induces a decrease in the damping peak and a shift towards higher temperatures. These results point out the effective stress transfers between the hemp fibres and the fully biobased epoxy system. The high mechanical properties and softening temperature measured in this work with a fully biobased epoxy system make this type of composite a very promising sustainable material for transport and lightweight engineering applications.

## 1. Introduction

With the successive oil crises and the current strong environmental concerns, the design and production of composites from renewable resources has become a major issue, which is especially crucial in the field of transport, mobilising numerous academic and industrial researchers. Renewable plant resources, such as starchy crops, lignocellulosic biomass and algae, are intensively studied to provide substitute routes to synthetic and petroleum-based compounds and to access to more sustainable molecules and materials [1,2,3]. This is particularly true for annual plant fibres such as flax and hemp, which exhibit high specific properties enabling them to compete with the glass fibres usually used in the field of composites [4,5,6]. To obtain competitive biobased high-grade composites, both the reinforcement and the matrix have to be wisely selected. Regarding the matrix, the use of thermosetting epoxy resins is common in industry for targeting structural applications owing to their high mechanical properties, temperature resistance and good adhesion to the reinforcement [7]. Up to now, bisphenol A (BPA)-based epoxy polymers, such as diglycidyl ether of bisphenol A (DGEBA), have provided more than 90% of the global production of epoxy resins [8,9]. However, the use of DGEBA presents the following two main drawbacks: (i) BPA used as synthesis precursor is exclusively petro-sourced, prepared from phenol; (ii) BPA is also considered as an endocrine disruptor [10], and its release from polymer resins has been clearly demonstrated [11].

Therefore, more sustainable and less toxic epoxy precursors are required. Thus, during the last years, research efforts have been devoted to obtaining epoxy monomers synthesised from biobased molecules [8,12,13,14,15,16]. To target high-performance composite applications, the resin must have a high elastic modulus and thus high T_g_ (glass temperature) values. From a chemical point of view, this can be achieved by using epoxy monomers and hardeners containing rigid aromatic rings. Thus, several studies focus on eugenol, a phenylpropene present in a large variety of plants such as clove trees [17]. This precursor can provide polyepoxide materials with elastic moduli in the range of 3 GPa, and T_g_ values higher than 100 °C were recorded [18,19,20,21]. However, as the availability of this resource (130,000 tons per year [22]) is insufficient to cover the composite demand, other compounds derived from lignin have been studied. Indeed, lignin is the second most abundant polymer in nature, with a worldwide production estimated at 100 million tons per year [23]. In addition, lignin is also a waste product of the paper and agricultural industry.

Among the synthons obtained from lignin, guaiacol and vanillin were used to develop biobased thermosets [24,25,26,27,28]. Recently a new synthon, iso-eugenol, has been obtained by the palladium-catalysed depolymerisation of lignin [29,30]. From this molecule, a diepoxy monomer has been synthesised, allowing for the preparation of biobased polyepoxyde [31,32,33]. The optimisation of the diepoxy prepolymer synthesis to decrease its environmental impact and make it possible to scale up the synthesis has led to the obtainment of a biobased diepoxy resin, BioIgenox, that has been cross-linked with camphoric anhydride or HHPA [34].

The purpose of this work is to develop a BioIgenox–hardener system that best meets the specifications imposed by the manufacture and subsequent production of a biobased high-performance plant fibre-reinforced composite. The main objective of this study is to characterise the rheology and kinetics of the epoxy system with a view to optimising the composite manufacturing process. Thus, various aspects must be considered:

(i) The epoxy–hardener mixture must be fluid enough to facilitate the impregnation step at a temperature for which the cross-linking remains slow, i.e., at a temperature well below the curing temperature. This condition should allow for a gel time compatible with conventional composite manufacturing processes. The cross-linking temperature should obviously be lower than the degradation temperature of natural fibres. Usually, the viscosity required for these systems should be less than 1 Pa s. for vacuum infusion.
(ii) The prepolymer mixture will be selected to give strongest fibre/matrix interface by maximising the attractive interactions, i.e., weak and, if possible, covalent bonds between the polymer matrix and the biochemical components of the fibre wall. Moreover, a system compatible with the water present in the plant fibres would be desirable in order to avoid the fibre dehydration step before the manufacture of the composite.
(iii) The reactivity, non-toxicity and biosourcing aspects will also be considered to select the optimal epoxy–hardener system.
(iv) The chemical structures of the prepolymers should provide the cross-linked material with sufficiently high mechanical properties for structural composite applications (E (Young modulus) > 2 GPa, σ_R_ (stress at failure) > 50 MPa, T_g_ > 100 °C…).

Thus, the first step of this study was to choose the hardener considering the different parameters previously listed. To answer this question, a study of the evolution of the viscosity of the BioIgenox–hardener mixture at different temperatures and over time as well as a study of the cross-linking reaction by DSC analysis were carried out. This first step has allowed us to define the conditions for the preparation of specimens made in neat polymer or in composites reinforced with unidirectional hemp fibres. The polyepoxide and composite specimens were then mechanically characterised using three-point bending tests and DMA tests. In parallel, as BPA is able to interact with several nuclear receptors as oestrogen, androgen and pregnane X receptors, the nuclear receptors [35,36], BioIgenox and its precursor have been evaluated using reporter cell lines expressing these nuclear receptors and compared with BPA and DGEBA.

## 2. Materials and Methods

**Figure 1:**
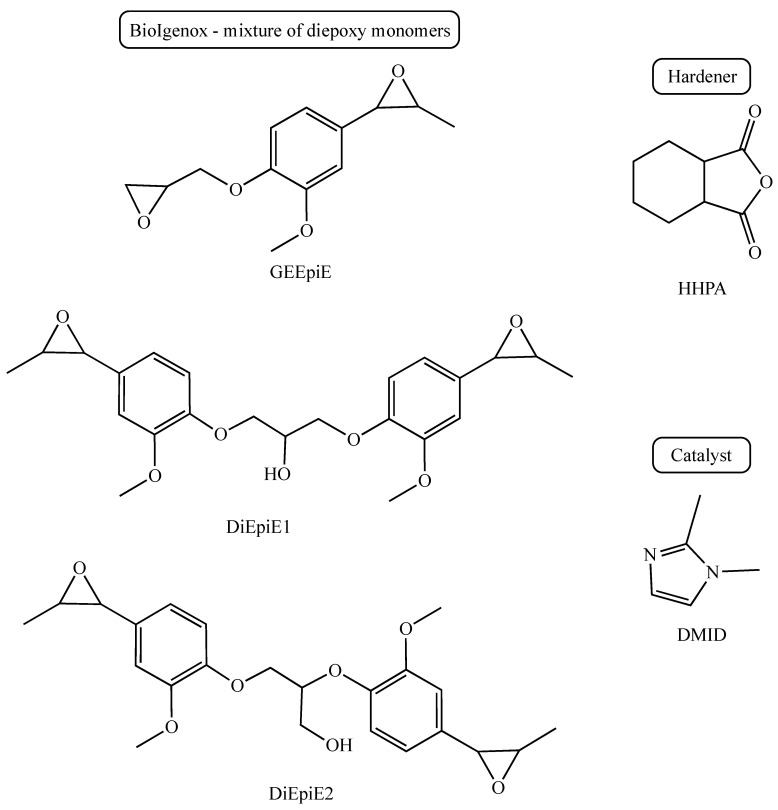
Molecular representations of the reagents.

BioIgenox resin, derived from isoeugenol, was prepared according to a published synthesis protocol [34]. Hexahydrophtalic anhydride (HHPA) and 1,2-dimethylimidazole (DMID) were purchased from Sigma-Aldrich (Gillingham, UK). Molecular representations of the reagents are depicted in Figure 1.

A 100% hemp quasi-unidirectional fabric made of low twisted rovings was used to manufacture the composites [37]. It was supplied by the company Linificio y Canapaficio Nazionale. The thin hemp twisted yarns present in the warp direction were removed before the composite production to obtain a pure unidirectional reinforcement.

The polymers and unidirectional composites were produced from the above-mentioned thermosetting epoxy system and hemp fibres in the form of square plates (10 × 10 cm^2^, 1 to 1.8 mm in thickness) using thermocompression. A PTFE-coated steel mould was used. The curing protocol included a first heating step at 70 °C for 1 min followed by a second step at 120 °C for 10 min. A post-curing (1 h at 120 °C) was applied after the unmoulding of the plates. For the neat epoxy plate, the weight fractions were 46.8%, 52.4% and 0.8% for BioIgenox, HHPA and DMID, respectively. For the composite plate, the fibre volume fraction was approximately 25% with a porosity level of 2.2%.

The DMA and bending specimens were then cut in the plates using a Trotec (SP500, Trotec Laser, Plymouth, MA, USA) laser cutting device. The specimens were stored for at least four weeks in a climatic chamber at 21 °C with 50%RH.

Rheological measurements were carried out using a thermoscientific Haake Mars (Modular Advanced Rheometer System) III equipped with a Peltier-controlled plate. A plate/plate measuring geometry with a diameter of 20 mm and a gap of 1 mm was used. Single-use plates were made of aluminium.

Differential scanning calorimetry (DSC) analyses were performed on a Perkin Elmer DSC under nitrogen flow (30 mL min^−1^) with a sample mass of 10 ± 3 mg. To study the curing reaction, samples were heated from 20 °C to 180 °C at a heating rate of 5 °C min^−1^. The determination of the glass transition temperatures (T_g_) was carried out on the samples cured in the oven. After a first heating from 20 °C to 180 °C, the T_g_ was determined on the second heating from 20 °C to 190 °C at a heating rate of 10 °C min^−1^ and using the pyris analysis software (version 12.1.0106).

A Metravib DMA+300 machine (Metravib, Limonest, France) was used to perform the DMA tests under a tensile–tensile loading configuration. For the composite samples, the length between the grips was 50 mm and the thickness and width were approximately 1.6 and 3.5 mm, respectively. For the neat resin specimens, the length between the grips was 40 mm, while the thickness and width were 1.7 and 10 mm, respectively. The frequency of the solicitation was swept between 1 and 40 Hz. A static displacement of 25 µm and a dynamic displacement of 8 µm were applied. The temperature sweep was stepped every 5 °C between 25 °C and 165 °C and up to 200 °C, depending on the specimens. The heating rate was 0.08 °C s^−1^. Three minutes of stabilisation were observed at each temperature plateau before starting the measurements. For each batch, two specimens were systematically tested. The storage modulus (*E*′), loss moduli (*E*″) and loss factor (tan*δ*) were determined for each temperature and frequency level. A very good reproducibility of the measurements was observed. For reasons of readability, in the present paper, the results are presented for one specimen only.

The polyepoxide and composite specimens were also characterised using 3-point bending tests on a universal testing machine, the MTS criterion 45, equipped with a 1 kN full-range load sensor. The tests were realised in accordance with the ASTM D790 standard. At least five samples of each batch were tested at a constant crosshead displacement rate of 1 mm/min. The deflection was measured at mid-span, on the bottom face of the specimen, using a micrometre laser sensor (the micro-epsilon optoNCDT 1420, Micro-Epsilon, Saint-Germain-en-Laye, France). The support span was 75 mm. The ultimate stress (σ_max_), the 3-point bending modulus (E) and the strain at failure (ε) were determined from the load–deflection curves using the following equations:

σ_max_ = (3F_max_ L)/2bh^2^ (1)

ε = 6hf/L^2^ (2)

where F_max_ is the maximum force applied to the sample, L is the span length, b and h are the width and thickness of the sample, respectively, and f is the deflection.

The 3-point bending modulus was determined using the slope of the stress–strain curve in the range of 0.1–0.3% of strain.

Toxicology: The agonist and antagonist nuclear receptor activities of BPA, DGEBA, BioIgenox, IE and GEIE were measured in bioluminescent reporter cell lines, as already described [35,38]. Briefly, HELN ERα, UALH AR and HG5LN GAL4-PXR reporter cell lines were seeded at a density of 25,000 cells per well in 96-well white opaque tissue culture plates (Greiner CellStar, Frickenhausen, Germany). Twenty-four hours later, the chemicals to be tested were added alone (agonist tests) or in the presence of 17 β-estradiol (E2), methyltrienolone (R1881) or SR12813 (antagonist tests) into the wells. Cells were incubated at 37 °C for 16 h. At the end of the incubation period, the culture medium was replaced with a medium containing 0.3 mM luciferin. Luciferase activity was measured for 2 s in intact living cells using a plate reader (PerkinElmer Luminometer, Waltham, MA, USA).

## 3. Results and Discussion

### 3.1. Choice of the Hardener

The biobased BioIgenox synthesis was optimised to both decrease its environmental impact and make it possible to scale up the synthesis [34]. This has led to the obtainment of the BioIgenox resin (BI) composed of a diepoxy monomer (GEEpiE) and dimers (DiEpiE), as depicted in Figure 1.

The ^1^H NMR analysis of the BI resin allowed for the easy quantification of the monomer and dimer molar ratios [34]. It was found that the monomer GEEpiE was the major compound obtained, and its molar ratio varied from 55% to 85%. It is interesting to note that the diepoxy BI resin contained both glycidyl ether oxirane functions (present only in the monomer GEEpiE) and non-glycidyl ether and internal oxirane functions, which were mostly present. Because of the mesomeric and inductive attractive effects due to the presence of the phenoxy group, the reactivity of these latter epoxydes will then be favoured by a Lewis acid group as anhydride hardeners [39]. Moreover, as reported by Paramarta and Webster [40], anhydride hardeners are also preferred to diamines for their higher reactivity in the case of internal epoxide function, which is less reactive compared to terminal epoxyde.

Moreover, the use of an anhydride hardener instead of an amine hardener has three important advantages:

(1) The cross-linking reaction is much less exothermic. This point is particularly interesting to make the temperature control easier during the implementation of the composite, and thus to prevent the damage to the natural fibres.
(2) The final cross-linked product is less sensitive to oxidation reactions [41].
(3) Lastly, the hydroxyl functions present at the fibre wall could also react with the anhydride functions, leading to the reinforcement of the fibre–matrix interface.

For these reasons, the anhydride hardener is preferred to the conventional toxic polyamine hardener for the cross-linking reaction of the BioIgenox resin.

Among the different anhydride hardeners, the choice of HHPA anhydride is based on several criteria:

(i) HHPA is a known solid hardener for epoxy resins. It contains a rigid ring that allows for the reinforcement of the mechanical properties of thermosets.
(ii) HHPA has a low melting point (37 °C) and, in addition, a low melt viscosity (47 cps at 40 °C). Thus, it allows us to obtain a liquid mixture with the epoxy resin at a low temperature and facilitates the thermoset processing.
(iii) With HHPA, the exothermic peak related to the cross-linking reaction is observed at about 100 °C, which is compatible with the use of the vegetable fibre reinforcements and limits the energy input during processing.
(iv) HHPA can be a biobased anhydride. Indeed, it can be prepared by the catalytic hydrogenation of phthalic anhydride [42], a molecule recently described as biobased [43].

### 3.2. Choice of the Composite Processing: DSC Analyses and Rheological Measurements

The conditions of the implementation of the composite must satisfy at least these three conditions: (i) the viscosity of the mixture must be compatible with conventional composite manufacturing processes, (ii) the cross-linking temperature must be lower than the degradation temperature of natural fibres, (iii) and the working time of the resin must be compatible with the time required for processing.

As depicted in Figure 1, BioIgenox resin typically contains a certain amount of hydroxyl groups that play an important catalytic role in the kinetics of the curing process, but which also provide a higher viscosity, which is obviously dependent on the number of hydroxyl functions and thus of the dimer/monomer ratio [39]. Consequently, the composition of the BI must be considered both to adapt the optimised BI-HHPA-DMID weight ratio, but also to define the most appropriate processing conditions in relation to the viscosity of the mixture and the cross-linking kinetics. For this study, the batch of BioIgenox used had a monomer/dimer molar ratio of 0.78/0.22, which was determined by NMR [34].

**Figure 2:**
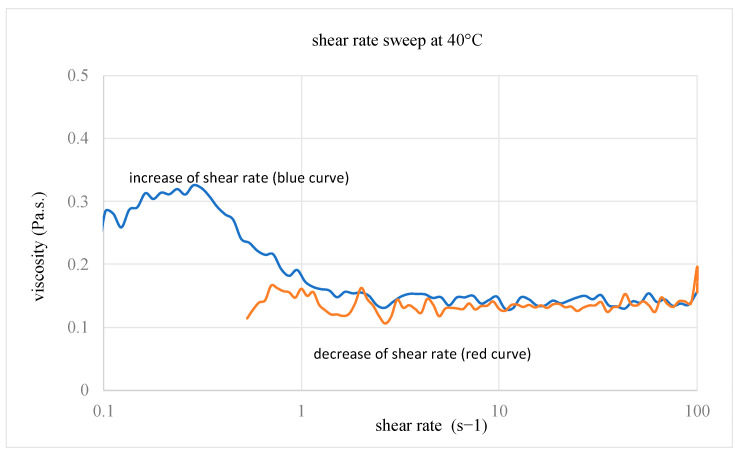
Evolution of the BI resin viscosity as a function of the shear rate.

As the melting temperature of the HHPA is 37 °C, the BI/HHPA/DMID mixture with a weight ratio of, respectively, 1/1.12/0.02, was prepared at 40 °C to be sufficiently fluid to obtain a homogeneous mixture. We can note that this mass ratio corresponds to a molar ratio epoxy function/anhydride function/DMID of 1/1/0.025. As depicted in Figure 2, the viscosity measurements carried out at 40 °C showed the Newtonian behaviour of this mixture in the range of 0.5–100 s^−1^ with a viscosity value of 0.14 ± 0.06 Pa s.

This viscosity value is thus compatible with the traditional implementation processes of the composites. Subsequently the viscosity measurements of the mixture were all performed with a shear rate of 1 s^−1^.

**Figure 3:**
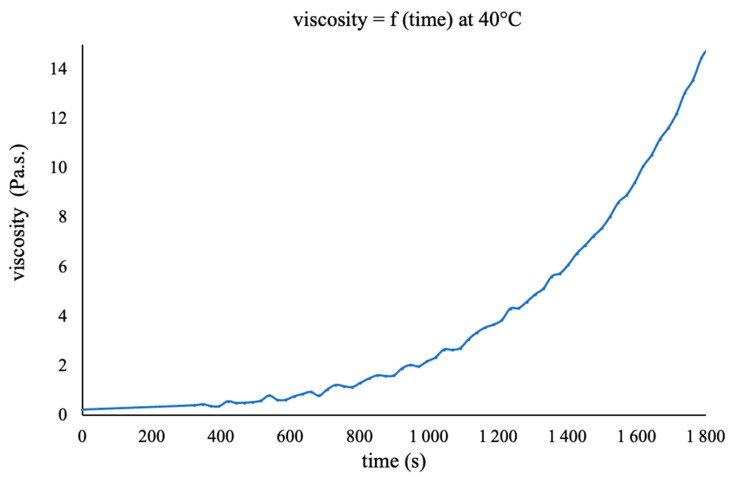
The evolution of the viscosity of the mixture of BI/HHPA/DMID with a 1/1.12/0.02 wt ratio at 40 °C as a function of the reaction time.

The evolution of the viscosity of the mixture at 40 °C as a function of time is shown in Figure 3.

The viscosity increases progressively with the cross-linking of the resin, remaining below 0.7 Pa s. during the first ten minutes, which is compatible with the time needed to prepare the composite. Obviously, these rheological measurements cannot be carried out in the presence of fibres; one can nevertheless think that the cross-linking can be slowed down in the presence of fibres because of the dilution induced by these fibres.

In parallel, a DSC study allowed us to define a temperature program for the curing of the epoxy resin during the manufacturing of the composite specimens. It is again important to bear in mind that the cross-linking kinetics can be significantly affected by the composition of the BI resin; indeed, the hydroxyl functions present in the dimers are known to catalyse the cross-linking reaction. Moreover, because of the exothermicity of the cross-linking reaction, it is often recommended to carry out a progressive rise in temperature in order to better control the reaction and to avoid local heating, which could damage the fibres and the material.

**Figure 4:**
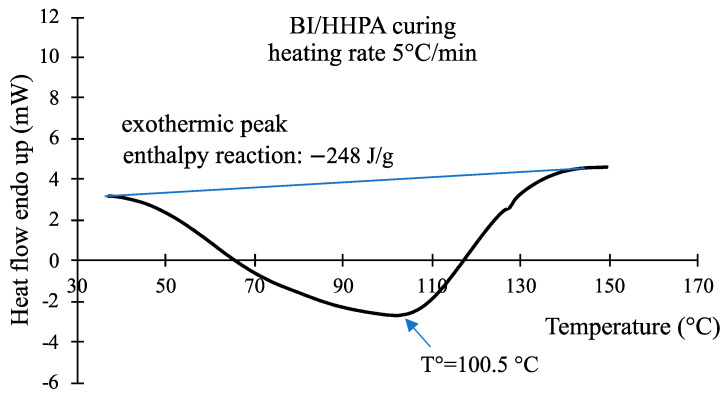
The study of the cross-linking reaction by the DSC, the blue line is used to measure the area of the exothermic peak.

To study the cross-linking reaction by the DSC, the sample was heated at 5 °C/min from 30 to 180 °C. It appears (Figure 4) that the cross-linking reaction starts at about 40 °C, with a maximum rate at about 100 °C, and this reaction releases approximately 250 J/g.

**Figure 5:**
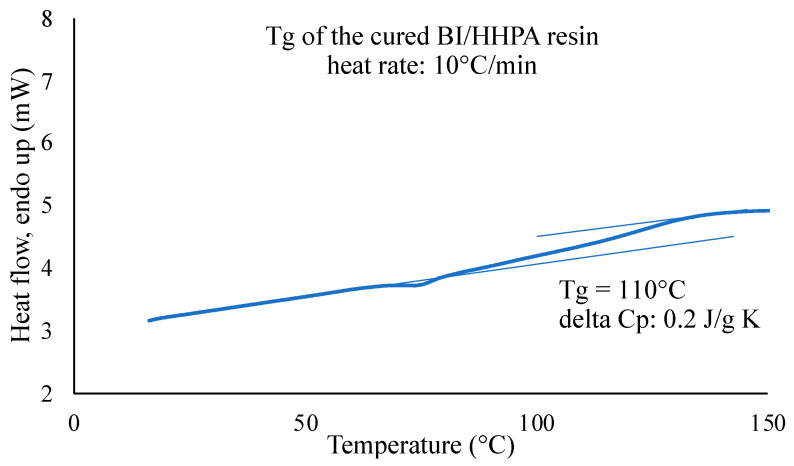
Determination of the T_g_ of the cross-linked BI/HHPA mixture using tangent method, the lines shown in light blue allow T_g_ to be determined using the tangent method.

The second heating of this sample (Figure 5) has allowed us to determine a T_g_ of about 110 °C.

From these data, we decided to start the cross-linking reaction at 70 °C and then to have a post-curing step at 120 °C, which is slightly higher than the T_g_, but not too high, thus avoiding a possible degradation of the natural fibres.

**Figure 6:**
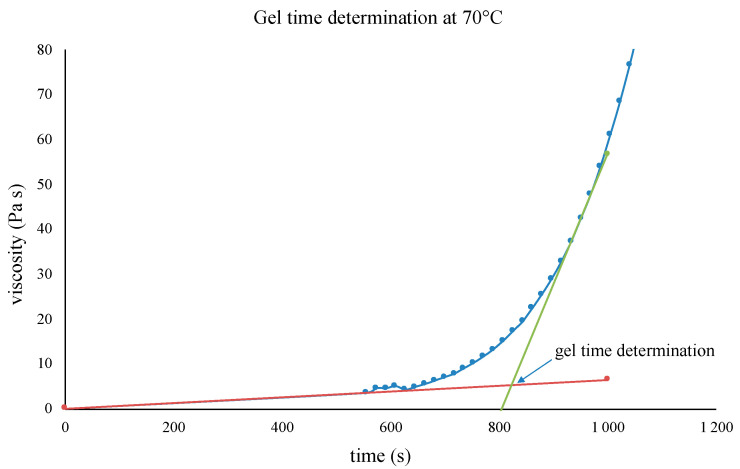
BI/HHPA system viscosity at 70 °C as a function of the cross-linking time, the colored lines (green and red) are the tengent used for the gel-time determination.

**Figure 7:**
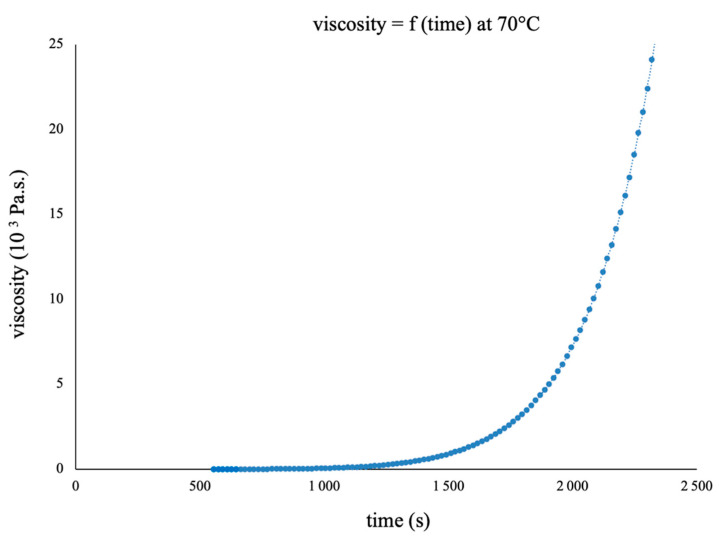
BI/HHPA system viscosity at 70 °C as a function of the cross-linking time.

Therefore, the evolution of the viscosity as a function of time has been measured at 70 °C. The strong increase in the viscosity appearing at ~12 min allows us to determine the gel time, while the viscosity continues to increase until a solid is obtained (Figure 6 and Figure 7). These data agree with the DSC analyses, which show that a sample cross-linked for 40 min at 70 °C is vitrified at an ambient temperature with a T_g_ of 57 °C.

Thus, the program of implementation of the selected composite will consist in impregnating the fibres at 35–40 °C; then, the mould is placed in an oven and the following temperature program is applied: 10 min at 70 °C, temperature ramp from 70 to 120 °C at 5 °C/min and 10 min at 120 °C.

### 3.3. Characterisation of the Final Material

#### Bending Behaviour

**Figure 8:**
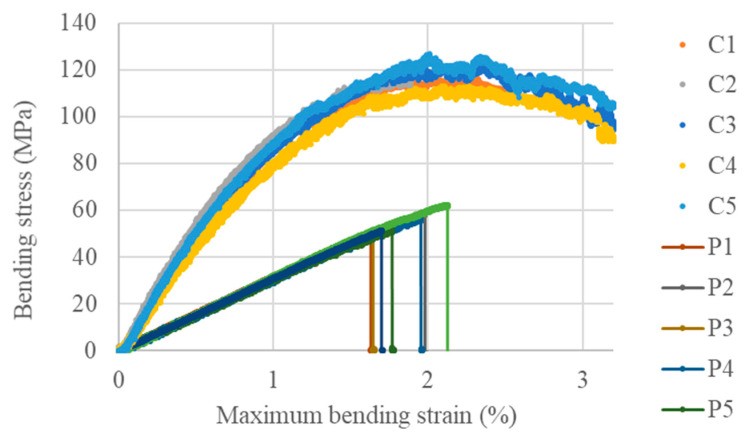
Three-point bending curves of the BI-HHPA epoxy polymer (P) and its hemp composite (C) (fibre direction).

The BI/HHPA polymer exhibits an elasto-brittle behaviour (Figure 8) characterised by an average bending modulus of 3.1 GPa and a maximum bending stress and strain of approximately 55 MPa and 1.82%, respectively (see Table 1). When reinforced with the UD hemp fibres, the bending properties improve significantly, reaching values in the fibre direction of 10.1 GPa, 125 MPa and 2.64% for the bending modulus, strength and strain at maximum stress, respectively. These values align with theoretical predictions based on a rule of mixture, considering the fibre volume fraction (25%) and the average elastic modulus and strength of the long hemp fibres, which are reported in the literature [44] to range between 45 and 64.3 GPa, and 318 and 616 MPa, respectively. Interestingly, it is observed that the composite exhibits less brittleness than the neat polymer. The inelastic behaviour can be attributed to the fibres themselves, which exhibit a non-linear behaviour in their longitudinal direction [45], or possibly to the increased mobility at the interface between the fibres and the epoxy polymer, as well as modifications in the cross-linking and 3D network of the epoxy polymer when cured in the presence of fibres.

**Figure 9:**
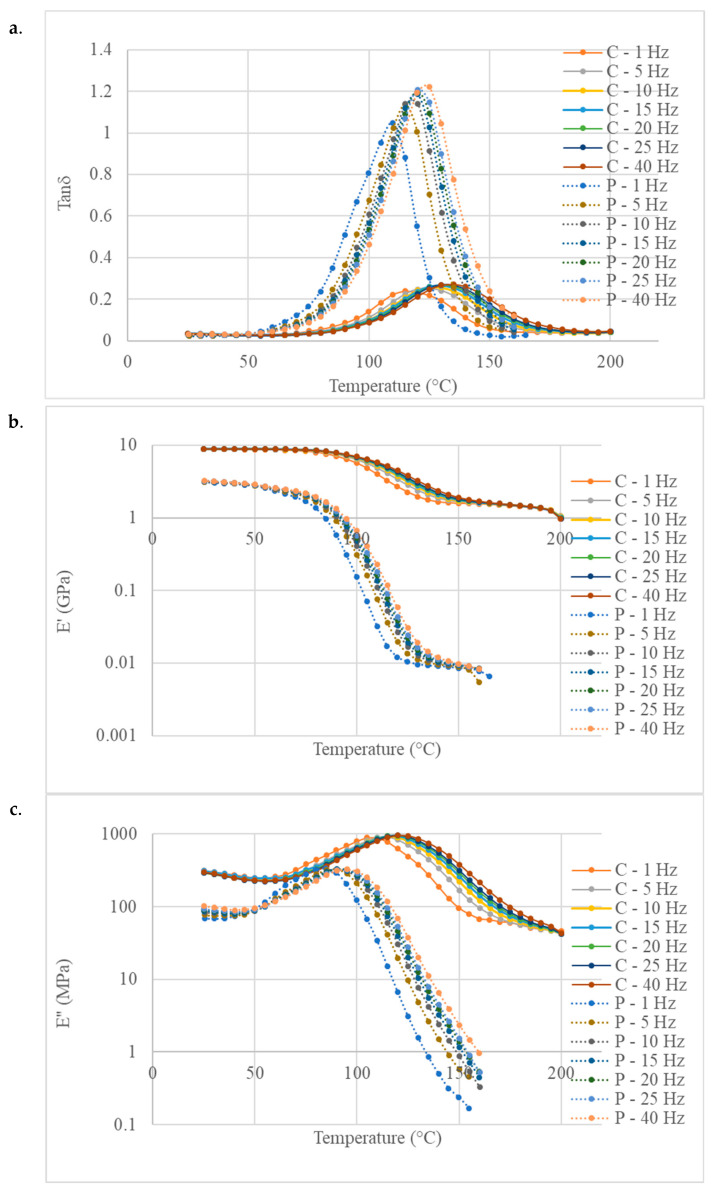
DMA curves of the BI-HHPA epoxy polymer (P) and its hemp composite (C) in the fibre direction: (**a**) the loss factor, (**b**) storage modulus and (**c**) loss modulus.

The DMA tests provide insight into the evolution of the viscoelastic properties of the polymer and its composite as a function of temperature across the various frequencies tested in this study (Figure 9). The storage modulus values at 25 °C align well with the elastic modulus values obtained through quasi-static three-point bending tests.

The following three different methods were used for the T_g_ determination from the DMA tests: the maximum value of the E′ curve derivative, the peak of the E″ curve and the peak of the tanδ curve. The values for a frequency of 1 Hz are summarised in Table 2. The values obtained from these methods do not precisely coincide. The DMA plots illustrate that the transition occurs over a temperature range. Typically, the values derived from the E″ peak provide a more consistent and appropriate indicator than those based on the tanδ peak. The E″ peak signifies the temperature at which the material undergoes a maximum change in the polymer chain mobility, while the tan*δ* peak characterises the material’s damping capacity. The determined values are approximately 85 °C for the polymer and 110 °C for the composite.

The softening temperature of the composite surpasses the T_g_ measured for the neat resin by approximately 25 °C. The influence of the fibres on the T_g_ has been previously observed and documented for plant fibre composites [46,47,48,49,50,51,52,53]. Oksman et al. [53] and Tajvidi et al. [46] observed a decrease in the T_g_ when adding fibres in PLA and PP polymers, respectively. Gupta et al. [52] demonstrated for jute fibres and epoxy resin that the change in the T_g_ can vary depending of the fibre volume fraction in the composite. The discrepancy between the T_g_ of the composite and that of the neat polymer is attributed to interfacial interactions between the polymer and fibres, which can also be affected by the presence of a plasticiser [51] or a sizing agent when used and/or changes in the cured resin’s structure around the fibres [50]. The introduction of fibres may affect the initial curing rate of the resin within the composite, potentially due to a localised increase in heat. Additionally, with plant fibres, some epoxy reagents may be adsorbed onto the fibre surface or absorbed into the fibre walls, altering the local concentrations and affecting the cross-link density at the fibre/matrix interface. This can result in both stiffening and softening effects adjacent to the interface due to curative depletion.

Similar effects leading to an increase in the T_g_ at the composite scale have also already been observed and depicted with epoxy systems and carbon nanotubes [50]. With plant fibres, Gupta and Srivastava [52] associated the shift in the T_g_ to higher temperatures, with decreased matrix mobility due to the incorporation of fibres.

Moreover, the increase in the T_g_ could be attributed to moisture introduced by the plant fibres during the epoxy curing. Liang Li et al. [54] suggested that water molecules forming double-hydrogen bonds with the polymer could elevate the T_g_ in the epoxy resin. Boutin et al. [55] also showed that the incorporation of flax fibres in the DGEBA/DETA matrix can modify the network formation, with water potentially altering the amine functions of the hardener, leading to a decrease in the matrix glass transition temperature (up to 40 °C). Conversely, the water added to the resin may accelerate the epoxy/amine reaction, slightly increasing the T_g_ of the matrix.

Figure 9 also shows a significant decrease in the damping capacity (tanδ at peak) when hemp fibres are added to the epoxy polymer. The values range between 0.22 and 0.26 compared to 1.05 and 1.22 for the neat epoxy. This phenomenon has been previously reported for petroleum-based epoxy with carbon fibres as well as plant fibres [46,56,57]. The incorporation of stiff fibres in epoxy resin restricts the movement of polymer chains, thereby reducing the height of the tanδ peak.

Both observations, namely, the reduction in the damping peak and its rightward shift for the composite material, indicate highly effective stress transfer between the fibre and the matrix.

This characteristic renders this type of composite a promising sustainable material for applications in transport and lightweight engineering.

### 3.4. Toxicology

A major concern in the development of new platform chemicals to be competitive with bisphenol A is the propensity of these chemicals to interact with the human nuclear receptors, and how this interaction compares to current bisphenol analogues. In this work, BioIgenox, IE and GEiE were compared to bisphenol A and DGEBA for their propensity to interact with ERα, AR and PXR.

The oestrogenic agonistic potential of the chemicals at 10 μM was monitored by using HELN hERα reporter cells (Supplementary Figure S1A). In these cells, bisphenol A (BPA) exerts a partial potency for luciferase activity (60% activity of the transactivation seen with estradiol (E2)). DGEBA, BioIgenox, IE and GEiE were demonstrated to be completely inactive. The antagonistic potential of the chemicals was also assessed in HELN ERα cells in the presence of E2 0.1 nM. All of the compounds were devoid of antagonistic activity, except for BPA, which partially inhibited luciferase expression (Supplementary Figure S1B).

The androgenic agonistic potential of the chemicals at 10 μM was also monitored on (Supplementary Figure S1C) the transcriptional activity by using U2OS hAR reporter cells [58]. All of the compounds were devoid of agonistic activity. The antagonistic potential of the chemicals was also assessed in U2OS hAR cells in the presence of R1181 0.3 nM. Except for BPA, all of the compounds were devoid of antagonistic activity (Supplementary Figure S1D).

The agonistic potential of the chemicals at 10 μM was also monitored on hPXR (Supplementary Figure S1E) transcriptional activity by using HG5LN GAL4-hPXR reporter cells [29]. Among them, BPA and GEiE were slightly active. All of the compounds were devoid of agonistic activity. The antagonistic potential of the chemicals was also assessed in HG5LN GAL4-hPXR cells in the presence of SR12813 0.3 μM. All of the compounds were devoid of antagonistic activity (Supplementary Figure S1F).

## 4. Conclusions

In this work, the production of totally biobased high-performance composites has been demonstrated. The described epoxy resin/hardener system meets the specifications imposed by composite processing conditions, i.e., a viscosity and gel time compatible with fibre impregnation.

The results collected in the present paper show that fully biobased and eco-friendly synthetised epoxy polymer can compete with the best petroleum-based fully established ones. The proposed BioIgenox/HHPA epoxy polymer has a bending modulus, a bending strength, a maximum strain at failure and a T_g_ of, respectively, 3.1 GPa, 55 MPa, 1.82% and 120 °C. All of these properties are consistent with its use as a matrix for structural plant fibre composites. Moreover, unlike BPA, these compounds do not interact with ERα, AR and PXR nuclear receptors, and are thus devoid of endocrine disruptor activities.

In order to choose the composite processing, DSC analyses and rheological measurements have been carried out. Based on these results, a composite processing conditions that prevent natural fibre damage have been developed.

The thermal and mechanical characteristics of the final material were studied. A significant increase in the bending properties was observed between the BI/HHPA polymer and the reinforced UD hemp fibre composite. Interestingly, it is observed that the composite exhibits less brittleness than the neat polymer. The incorporation of fibres into the fully biobased epoxy system induces a decrease in the damping peak and a shift towards higher temperatures. A softening temperature of 110 °C is obtained at the scale of the composite material. These results point out the effective stress transfers between the hemp fibres and the fully biobased epoxy system.

This study demonstrates the potential of fully biobased composites for semi-structural applications compatible with conventional composite manufacturing processes. The next step will be dedicated to the transition from a laboratory scale to small-scale production.

## Figures and Tables

**Table 1:** Three-point bending properties of BI-HHPA epoxy polymer and its hemp composite in the fibre direction.

Mean Value ± Standard Deviation	Bending Modulus (GPa)	Bending Strength (MPa)	Maximum Bending Strain (%)
Polymer (BI-HHPA-DMID)	3.1 ± 0.08	54.8 ± 4.6	1.82 ± 0.19
Composite	10.1 ± 0.66	125.4 ± 15.6	2.64 ± 0.78

**Table 2:** Glass transition temperatures of the epoxy polymer and the hemp/epoxy composite determined by the DMA from the maximum value of the E′ curve derivative, the peak of the E″ curve and the peak of the tanδ curve (frequency: 1 Hz).

Glass Transition Temperature (T_g_) in °C	Polymer (BI-HHPA-DMID)	Composite
E′ max derivative	100	115
E″ peak	85	110
tanδ peak	110	115

## Data Availability

The original contributions presented in the study are included in the article/Supplementary Material, further inquiries can be directed to the corresponding author/s.

## Supplementary Materials

The following supporting information can be downloaded at: https://www.mdpi.com/article/10.3390/polym16142010/s1, Figure S1: title ERα, AR and PXR agonistic and antagonistic activities of BPA, DGEBA, BioIgenox, IE and GEiE in reporter cell lines.
