# Supplementary material for: Toward the Manufacturing of a Non-Toxic High-Performance Biobased Epoxy–Hemp Fibre Composite
Source: Polymers (Basel). 2024 Jul 13;16(14):2010. doi: 10.3390/polym16142010 (PMC11280780; doi:10.3390/polym16142010)
Supplement: Supplementary file 1 [file polymers-16-02010-s001.zip › polymers-3087123-supplementary.pdf]

Supplementary material

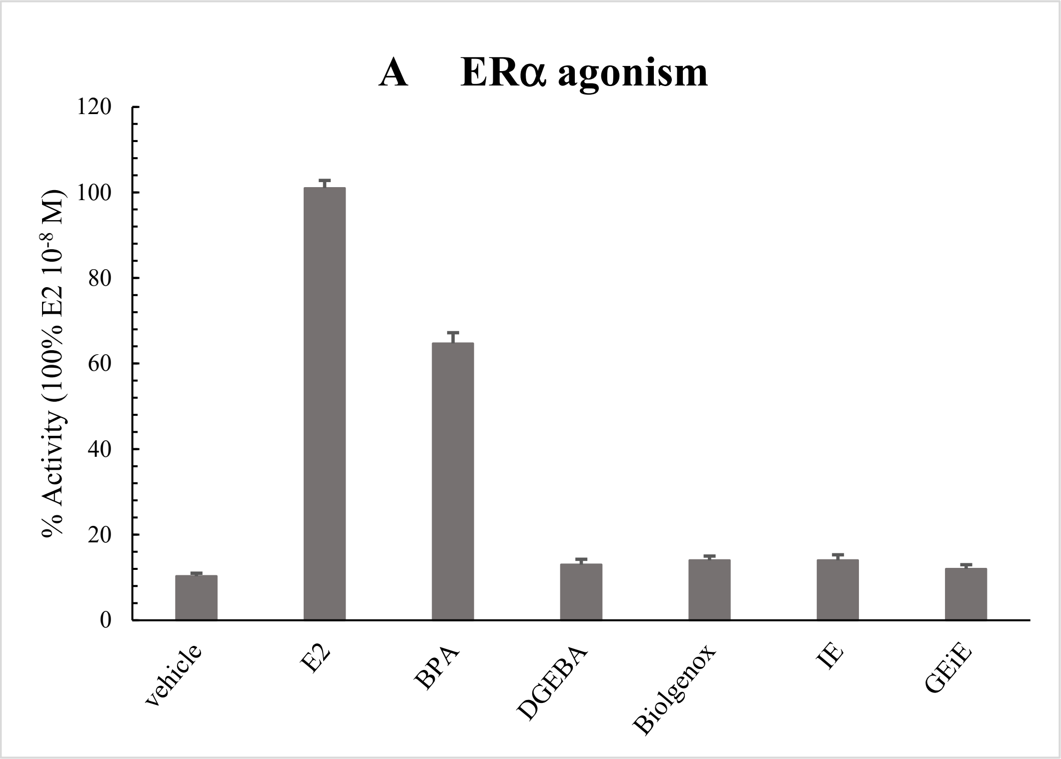

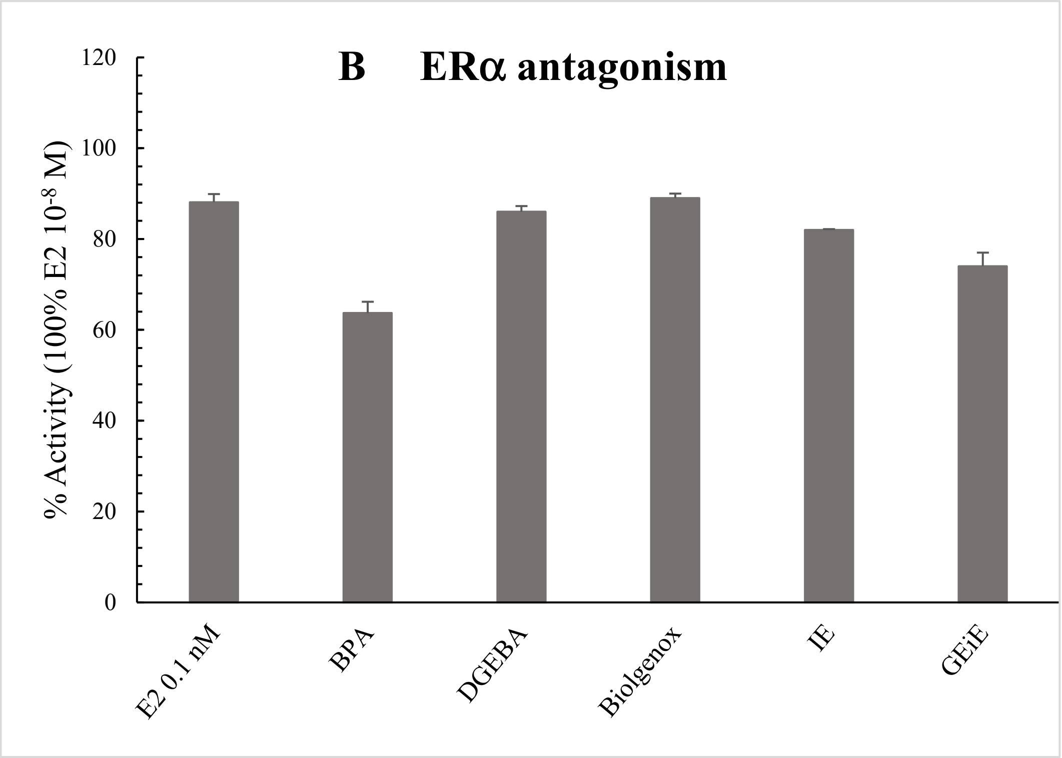

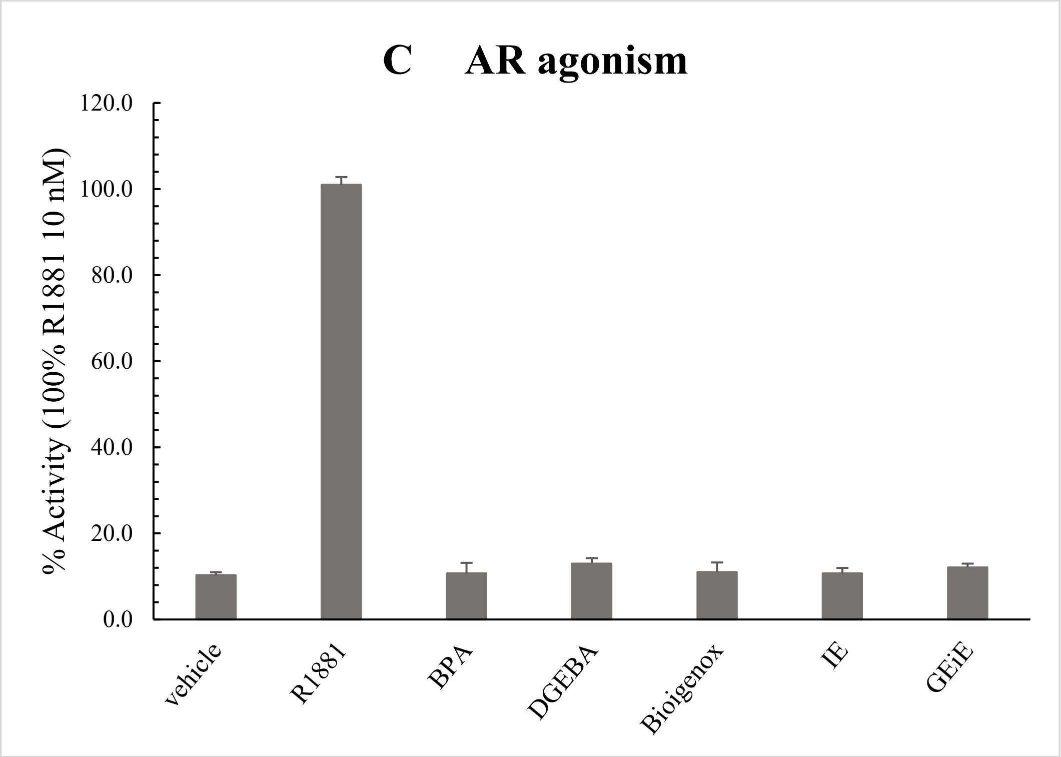
C AR agonism
% Activity (100% R1881 10 nM)
120.0
100.0
80.0
60.0
40.0
20.0
0.0
vehicle
R1881
BPA
DGEBA
Biolgenox
IE
GEiE

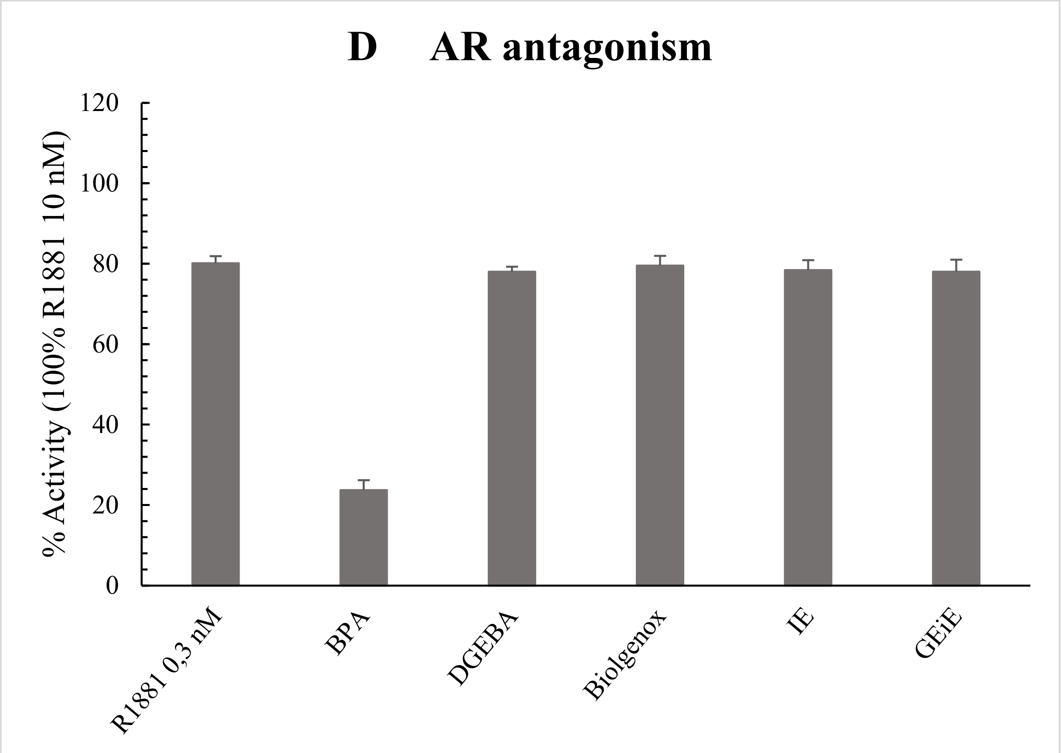
D AR antagonism
% Activity (100% R1881 10 nM)
120
100
80
60
40
20
0
R1881 0,3 nM
BPA
DGEBA
Biolgenox
IE
GEiE

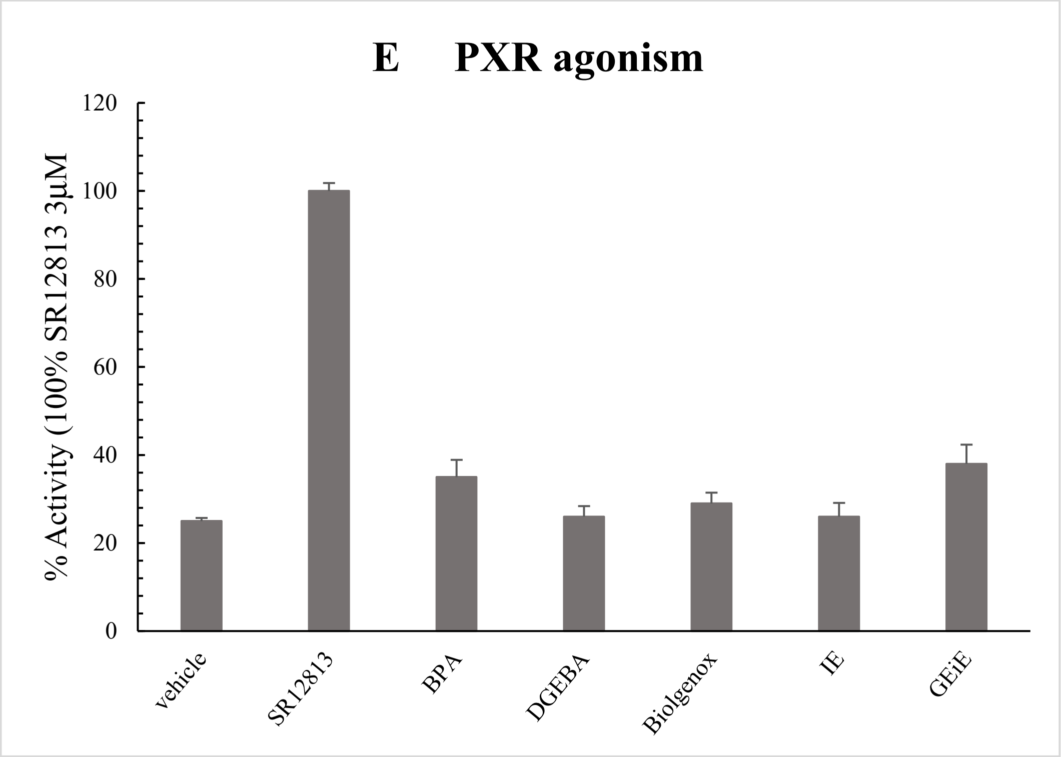

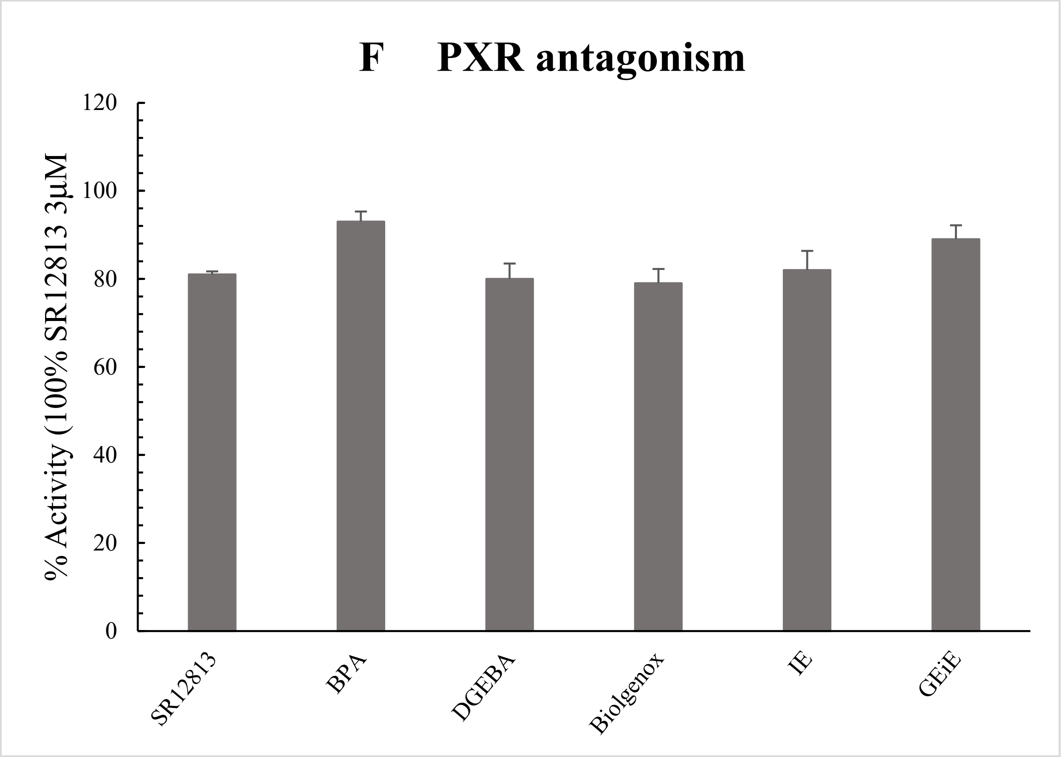

Figure S1: ERα, AR and PXR agonistic and antagonistic activities of BPA, DGEBA, BioIgenox, IE and GEiE in reporter cell lines. (A) ERα agonistic activity of chemicals. The HELN ERα cells were incubated for 16h with E2 (10 nM) or BPA, DGEBA, BioIgenox, IE and GEiE at 10 µM. (B) ERα antagonistic activity of chemicals. The HELN ERα cells were incubated for 16h with BPA, DGEBA, BioIgenox, IE and GEiE at 10 µM in presence of 0.1 nM E2. (C) AR agonistic activity of chemicals. The U2OS hAR cells were incubated for 16h in presence of 10 nM R1881 or BPA, DGEBA, BioIgenox, IE and GEiE diepoxy at 10 µM. (D) AR antagonistic activity of chemicals. The U2OS hAR cells were incubated for 16h

with BPA, DGEBA, BioIgenox, IE and GEiE at 10 µM in presence of 0.3 nM R1881. (E) PXR agonistic activity of chemicals. The HG5LN GAL4-hPXR cells were incubated for 16h with SR12813 (3 µM) or BPA, DGEBA, BioIgenox, IE and GEiE at 10 µM. (f) PXR antagonistic activity of chemicals. The HG5LN GAL4-hPXR cells were incubated for 16h with BPA, DGEBA, BioIgenox, IE and GEiE at 10 µM in presence of 0.3 µM SR12813. The maximal luciferase effect was obtained with 10 nM E2 (A) and (B), 10 nM R1181 (C) and (D) and 3 µM SR12813 (E) and (F). Values are the mean ± SD from three separate experiments.
